# 584. Phase 1 Placebo-Controlled Trial of COVI-VAC™, an Intranasal, Live Attenuated COVID-19 Vaccine

**DOI:** 10.1093/ofid/ofab466.782

**Published:** 2021-12-04

**Authors:** Sybil Tasker, Daryl Bendel, Melissa Bevan, Steffen Mueller, Anna Kushnir, Brandon Londt, J Robert Coleman

**Affiliations:** 1 Codagenix Inc., Farmingdale, New York; 2 hVivo, London, UK

## Abstract

**Background:**

COVI-VAC^TM^ is an intra-nasal live-attenuated SARS-COV-2 synthetic viral vaccine being developed for the prevention of COVID-19. COVI-VAC is attenuated through deletion of the furin cleavage site and introduction of 283 silent deoptimizing mutations that maintain viral amino acid sequence but result in significant attenuation due to slow translation in the human host cell. Notably, COVI-VAC includes all viral antigens and is not limited to spike. COVI-VAC has demonstrated attenuation, immunogenicity and single dose protection in both Syrian golden hamster and non-human primate models.

**Methods:**

48 healthy young adults were enrolled in an inpatient quarantine setting to one of 3 dose escalating cohorts and randomized to COVI-VAC or saline placebo given as nose drops, as a single 0.5mL dose or 2 doses 28 days apart. Endpoints included solicited and unsolicited adverse events, serum cytokines, viral shedding and sequence stability, mucosal and serum antibody responses and IFN ELISpot. Subjects will be followed for 1 year for late safety events and durability of immune response.

**Results:**

Dosing is complete. There has been no trend in solicited reactogenicity events, and all unsolicited adverse events reported to date have been mild. There have been no SAEs or Grade 3 or 4 events. Vaccine virus from anonymized subjects was shed at levels lower than that likely to result in onward transmission, and the deoptimized sequence of the shed virus remained unchanged compared to the original vaccine sequence. Unblinded data including immunogenicity will be available prior to the IDWeek meeting.

**Conclusion:**

COVI-VAC appears safe and well tolerated in healthy young adults. Vaccination resulted in minimal viral shedding without sequence instability. Safety and shedding data supports continued development in a wider Phase 2/3 population.

**Disclosures:**

**Sybil Tasker, MD, MPH, FIDSA**, **Codagenix Inc** (Employee, Shareholder) **Daryl Bendel, MD**, **Codagenix Inc** (Scientific Research Study Investigator) **Melissa Bevan, MD**, **Codagenix Inc** (Scientific Research Study Investigator) **Steffen Mueller, PhD**, **Codagenix Inc** (Board Member, Employee, Shareholder) **Anna Kushnir, PHD**, **Codagenix Inc** (Employee) **Brandon Londt, PhD**, **Codagenix Inc** (Other Financial or Material Support, contracted lab services) **J. Robert Coleman, PhD**, **Codagenix Inc.** (Board Member, Employee, Shareholder)

